# Protocol for a systematic review of mental healthcare for Afghan refugees and asylum seekers in high-income countries

**DOI:** 10.1136/bmjopen-2026-118502

**Published:** 2026-06-30

**Authors:** Sreeja Daliparthy, Faissal Sharif, Mohammad Sharif Razai

**Affiliations:** 1 Department of Public Health and Primary Care, University of Cambridge, Cambridge, UK; 2 Nuffield Department of Clinical Neurosciences, University of Oxford, Oxford, UK; 3 Primary Care Unit, Department of Public Health and Primary Care, University of Cambridge, Cambridge, UK

**Keywords:** Refugees, Psychosocial Intervention, MENTAL HEALTH

## Abstract

**Introduction:**

In the past five decades, Afghanistan has experienced one of the world’s largest and most protracted displacement crises. Stressors experienced during the pre-migration, transit and post-resettlement stages of asylum-seeking and refugee journeys can trigger or exacerbate adverse mental health outcomes. More than 750 000 Afghan refugees and asylum seekers reside in high-income countries (HICs), yet no recent review has synthesised evidence on their mental health status, barriers and enablers to well-being and accessing mental healthcare, and interventions and strategies to improve mental health outcomes in this population. This protocol describes a systematic review of literature reporting on the mental health experiences, barriers to and facilitators of mental well-being and interventions aimed at improving mental health outcomes among Afghan refugees and asylum seekers living in HICs.

**Methods and analysis:**

The Preferred Reporting Items for Systematic Reviews and Meta-Analyses (PRISMA) 2020 guidelines will be followed. MEDLINE, Embase, Web of Science, Cochrane Library, CABI Global Health and APA PsycINFO databases will be searched using keywords and Medical Subject Headings terms related to the research aims, developed and refined with the support of a medical librarian. Studies will be selected through duplicate title and abstract screening followed by full-text review against predefined eligibility criteria. Data extraction will use a structured template created for this review. A mixed-methods synthesis will be undertaken, with descriptive statistics used to summarise quantitative findings and narrative synthesis used to capture qualitative data. Findings will be integrated and analysed by subgroups where appropriate to account for expected heterogeneity in the data. Study quality will be appraised using the Mixed Methods Appraisal Tool.

**Ethics and dissemination:**

Ethical approval is not required, as no primary data will be collected. Findings will be disseminated through open-access peer-reviewed publications and conference presentations.

**PROSPERO registration number:**

CRD420261337124

**STRENGTHS AND LIMITATIONS OF THIS STUDY**

- Integrating qualitative, quantitative and mixed-methods evidence facilitates the capturing of reported mental health conditions, interventions, barriers to access and contextual enablers of care.
- A mixed-methods synthesis approach allows intervention findings to be interpreted alongside lived experiences and service pathways, which is relevant for displaced populations navigating complex and unfamiliar healthcare systems.
- Restriction to high-income host settings improves contextual coherence and supports more meaningful interpretation for policy and service delivery in these environments.
- Heterogeneity in study design and outcome measurement may limit comparability and quantitative synthesis.
- Restriction to high-income host countries may limit transferability to low-income and middle-income settings.

## Introduction

### Rationale

 Decades of armed conflict, foreign invasions, climate-related extreme weather events, chronic poverty and food insecurity and the recent systematic exclusion of marginalised groups, particularly women, from public life by Afghanistan’s de facto authorities have converged to produce a multidimensional crisis of which displacement is a chief consequence.^1^,^6^ An estimated 5.1 million Afghans worldwide are currently registered as refugees or asylum seekers,^7^ representing approximately 10.3% of the country’s total population of around 49.5 million.^8^ Most have sought refuge in neighbouring Iran and Pakistan^7^; however, both countries have recently initiated large-scale return processes—in many cases involuntary—thereby placing these populations at increased risk.^9^

Alongside displacement within the regions of West and South Asia, a substantial number of Afghan refugees have resettled in high-income countries (HICs), primarily across Europe and North America, including Germany, France, the United Kingdom and the United States.^9^ As of 2025, more than 750 000 registered Afghan refugees and asylum seekers reside in HICs.^7^

Research indicates that refugees and asylum seekers experience psychological disorders such as depression, anxiety and post-traumatic stress disorder (PTSD) at higher prevalence than the general population, with these often co-occurring conditions persisting for years after resettlement.^10^,^15^ Each step of the refugee experience—including pre-migration, displacement, transit, resettlement and integration—introduces stressors that may trigger or exacerbate mental health difficulties in these populations.^16^ Pre-migration traumas include poverty, persecution, exposure to violence or natural disasters and limited access to education or employment.^16^,^22^ The migration process itself can involve violence, detention, exposure to life-threatening transit conditions and lack of access to services for basic needs.^16^,^22^ Post-migration challenges may include unemployment, uncertain legal status, poor living or working conditions, difficulties navigating administrative systems, separation from support networks, exclusion within the host population and language and cultural barriers affecting integration.^16^,^22^

Although several studies have examined the prevalence of psychological disorders among refugee and asylum-seeking populations, barriers to accessing mental healthcare following resettlement and the effectiveness of different intervention types,^11^,^1517 18 20 21 23^ the evidence base remains fragmented. Some studies focus exclusively on HICs,^1030^,^32^ whereas others focus on refugees of Afghan origin^1933^,^36^; however, no recent reviews are oriented towards displaced Afghans living in HICs. A 2013 mixed-methods systematic review synthesised literature on psychological distress among Afghan refugees in industrialised countries and associated risk and protective factors,^37^ but did not address mental health interventions or assess their effectiveness. A systematic review in 2023 synthesised evidence-based mental health interventions for Afghan refugees, with a focus on cultural adaptation.^33^ Our review focuses on high-income host countries and is not limited to intervention studies. We will synthesise evidence on reported mental health conditions, prevalence estimates, risk and protective factors, mental healthcare access and intervention evidence.

Reviews with broader scopes draw on evidence from diverse contexts across different population groups, host countries and time since initial displacement. These contextual differences influence mental health outcomes and intervention effectiveness,^13 16^ with psychological disorder prevalence estimates varying by country of origin and host country–income level,^11^ making it difficult to extrapolate findings specifically to displaced Afghans living in high-income settings. Taken together with the fragmented and limited synthesis of existing evidence on the mental health needs of Afghan refugees and asylum seekers living in HICs, there remains a need to bring together evidence on barriers to and facilitators of mental healthcare, as well as interventions for improving mental well-being in this population. Furthermore, the mental health of Afghan nationals resettled in HICs must be studied in the context of imperial interventions, colonial legacies and racialised global power relations, particularly given the involvement of many high-income host countries in the decades-long US-led invasion and occupation of Afghanistan.^17 38^,^40^ At the same time, displacement and mental health outcomes are determined by multiple overlapping drivers, including ongoing violence, repression and governance under the Taliban, sex-based discriminatory policies and post-resettlement conditions in host countries.^16^,^2241^

### Objective

This article describes the protocol for a mixed-methods systematic review of evidence on the mental health of Afghan refugees and asylum seekers living in HICs. The review will synthesise evidence on reported mental health conditions and prevalence estimates; risk and protective factors at individual, interpersonal, community and structural levels for mental health; barriers to and enablers of mental healthcare access and use; and interventions designed to improve mental health outcomes in this population.

## Methods and analysis

### Reporting guidelines

This systematic review protocol follows the Preferred Reporting Items for Systematic Review and Meta-Analysis Protocols (PRISMA-P) guidelines.^42^ The review will be conducted and reported in accordance with the PRISMA 2020 statement, including searches of databases, registers and other sources.^43^ The protocol is registered with the International Prospective Register of Systematic Reviews (PROSPERO) CRD420261337124.

### Research questions

The review will address the following research questions: (1) which mental health conditions are reported in this population, and what prevalence estimates are available? (2) What are the risk factors for poor mental health in this population? (3) What individual, interpersonal, community, service-level and structural protective factors support mental well-being and resilience? (4) What barriers to, and enablers of, access to and use of mental health services are reported? (5) What interventions have been used to improve mental health outcomes in this population, and what evidence exists regarding their acceptability and effectiveness?

### Eligibility criteria

In this review, a *refugee* is defined as an individual who has been formally granted international protection under the 1951 Refugee Convention due to a well-founded fear of persecution, conflict or violence.^44^ An *asylum seeker* is an individual who has sought international protection but whose claim for refugee status has not yet been determined.^45^ *Afghan* is an individual who is a national of Afghanistan and whose country of origin is Afghanistan.

This review will include articles reporting on the mental health of Afghan refugees and/or asylum seekers in HICs, as described in table 1. The review questions and search strategy were developed using the Sample, Phenomenon of Interest, Design, Evaluation and Research type framework, as it is suited to reviews incorporating qualitative, quantitative and mixed-methods evidence.^46^ Studies to be included in the data synthesis will be full-text peer-reviewed articles and relevant open-access grey literature published in English. Eligible articles will include studies reporting mental health experiences, prevalence estimates for psychological conditions, risk and protective factors, barriers to and enablers of mental healthcare access and use, and the implementation, acceptability or effectiveness of interventions to improve mental health outcomes.

**Table 1:** Studies to be included in the review in the SPIDER format

Eligible studies	
Sample	Afghan refugees and/or asylum seekers in high-income countries
Phenomenon of Interest	Mental health conditions and psychological distress Experiences of mental well-being and mental ill-health Barriers to and enablers of accessing mental healthcare Mental health interventions (eg, clinical, community-based, digital and culturally adapted)
Design	Qualitative studies (eg, interviews, focus groups and ethnography) Quantitative observational studies (eg, cross-sectional and cohort) Interventional studies (eg, RCTs and quasi-experimental designs) Mixed-methods studies
Evaluation	Reported mental health outcomes (eg, depression, anxiety and PTSD) Self-reported experiences of mental well-being or distress Access, utilisation, acceptability and perceived effectiveness of services or interventions; identified barriers and facilitators
Research type	Qualitative Quantitative Mixed methods

PTSD, post-traumatic stress disorder; RCTs, randomised controlled trials; SPIDER, Sample, Phenomenon of Interest, Design, Evaluation, Research.

Studies that were not conducted in high-income economies for the 2026 fiscal year as defined by the World Bank, that is, countries with a gross national income (GNI) per capita above US$13 935,^47^ will be excluded from the synthesis. Moreover, any studies that are not focused on Afghan refugees and/or asylum seekers or studies that include Afghans but only report aggregated data for multiple refugee and asylum-seeking populations will also be excluded.

### Information sources

A comprehensive search was conducted on 15 January 2026 in MEDLINE via Ovid, Embase via Ovid, Web of Science, Cochrane Library, CABI Global Health and APA PsycINFO databases. Full-text review, data extraction, quality appraisal and synthesis are planned for completion by August 2026. Reference lists of included articles and any identified review articles will also be examined via bidirectional citation chaining. Relevant grey literature will be identified using Google Scholar and the websites of organisations such as the WHO and Médecins Sans Frontières.

### Search strategy

The search strategy was formulated and tested by the research team and iteratively refined in collaboration with a medical librarian (online supplemental appendix 1). Search terms incorporated database-specific subject headings (eg, Medical Subject Headings and Centre for Agriculture and Bioscience International terms) and natural language keywords. The strategy included three domains: mental health and psychosocial well-being, Afghanistan or Afghan identity and refugee or asylum-seeker status. The mental health and psychosocial well-being domain included two groups of terms: first, clinical and symptom-related terms such as depression, anxiety, PTSD, psychological distress and mental health; and second, experiential and service-access terms such as experiences, perceptions, attitudes and perspectives. These experiential terms were included to identify qualitative and mixed-methods studies that may describe mental well-being, distress, or access to care without using diagnostic terminology in the title or abstract. These terms were not treated as mental health conditions. Records in which experiences or perceptions are unrelated to mental health or mental healthcare will be excluded during duplicate screening.

The HIC inclusion criterion will be evaluated on a study-by-study basis during title and abstract screening to ensure that no studies conducted in HICs are missed because of restrictive search terms. Searches were limited to publications published between January 2001 and January 2026.

### Study selection

Identified records will be uploaded into Covidence systematic review software^48^ for record management. Titles and abstracts will be screened in duplicate against the eligibility criteria by two independent reviewers (SD, LBP and SA). Disagreements will be resolved through discussion or by consultation with a third reviewer (MSR). Selected records will then undergo duplicate full-text review by SD, LBP and MSR until the final set of articles for inclusion in the synthesis is determined. The study selection process will be reported using a PRISMA flow diagram in the final review, and the PRISMA reporting checklist will be completed and provided as online supplemental material.

### Data extraction

Data will be extracted from studies meeting the inclusion criteria. SD and MSR will extract data in duplicate from an initial sample of articles to refine the data extraction template; SD will then conduct data extraction for the remaining articles with oversight and review from MSR. Data extraction will include the information summarised in table 2. The extraction template may be refined iteratively if additional relevant data items emerge during the review process.

**Table 2:** Data extraction plan

Category	Variables to be extracted
Basic descriptive information	Title, first author(s), study year(s), publication reference
Contextual details	Host country, study type (eg, qualitative, quantitative and mixed methods), study design, focus of study (eg, mental health experiences, intervention(s) or access to care (or combinations of these themes)) and intervention details, if any (eg, name, description and delivery)
Research question(s)	Primary aims and secondary objectives
Participant characteristics	Whether refugees, asylum seekers or both are involved in the study, if any subgroups are the focus of the study (eg, Afghan refugees and/or asylum seekers recruited from specific ethnic groups, sexes and/or age ranges)
Outcomes	Reported measures, timepoints and analyses

### Outcomes and data synthesis

Given the underexplored nature of the mental health needs and access to services of Afghan refugees and asylum seekers in HICs, all eligible study designs will be included in the synthesis. For studies reporting mental healthcare interventions, outcomes relating to mental health experiences will be extracted, including any results related to psychological, behavioural and cognitive well-being, in addition to findings on risks and protective factors and experiences of navigating care pathways. Owing to the anticipated heterogeneity in study designs, populations and outcome measures, quantitative findings will be summarised using descriptive statistics only, and no meta-analysis will be undertaken. Findings will be synthesised through narrative synthesis, with themes and patterns identified across studies. Outcome reporting may be refined iteratively as the evidence base is reviewed.

Qualitative data on mental health experiences, barriers to care and enablers of mental well-being will be analysed using thematic analysis. Themes will be developed in relation to the research questions and used to compare findings across studies.

Quantitative and qualitative findings will be integrated during interpretation, allowing reported intervention outcomes to be considered alongside lived experiences, access to services and various contextual factors. Where relevant, findings will be analysed by subgroup (eg, host country or type of intervention) to support interpretation within high-income healthcare settings. The synthesis strategy will continue to develop based on the findings and type of data collected.

### Assessment of study quality and bias

Methodological quality will be appraised using the Mixed Methods Appraisal Tool (MMAT) version 2018.^49^ The MMAT includes criteria for qualitative studies, randomised controlled trials, non-randomised studies, quantitative descriptive studies and mixed-methods studies and is therefore appropriate for the range of designs expected in this review. Two reviewers will independently apply the relevant MMAT criteria for each study design. Disagreements will be resolved through discussion or, where needed, consultation with a third reviewer. In line with MMAT guidance, item-level ratings will be reported rather than a single overall numerical score.^49^ Study quality will be considered during interpretation, and findings from studies at higher risk of bias will be interpreted with appropriate caution.

### Patient and public involvement

Neither patients nor the public were involved in the design, conduct, reporting or dissemination plan of this research.

## Ethics and dissemination

This systematic review does not require research ethics approval because it will use published literature and will involve no participant contact or collection of new primary data. Findings will be disseminated through publication in an open-access peer-reviewed journal and presentations to academic, clinical, policy and refugee health audiences.

## Supplementary material

10.1136/bmjopen-2026-118502 online supplemental file 1

10.1136/bmjopen-2026-118502 online supplemental file 2
