# Supplementary material for: Protocol for a systematic review of mental healthcare for Afghan refugees and asylum seekers in high-income countries
Source: BMJ Open. 2026 Jun 30;16(6):e118502. doi: 10.1136/bmjopen-2026-118502 (PMC13330941; doi:10.1136/bmjopen-2026-118502)
Supplement: Supplementary file 3 [file bmjopen-2026-118502.reviewer_comments.pdf]

PEER REVIEW HISTORY

BMJ Open publishes all reviews undertaken for accepted manuscripts. Reviewers are asked to complete a checklist review form and are provided with free text boxes to elaborate on their assessment. These free text comments are reproduced below.

ARTICLE DETAILS

Title (Provisional)

Protocol for a Systematic Review of Mental Healthcare for Afghan Refugees and Asylum Seekers in High-Income Countries

Authors

Daliparthy, Sreeja; Sharif, Faissal; Razai, Mohammad Sharif S

VERSION 1 - REVIEW

**Reviewer**	**1**
**Name**	**Park, Soim**
**Affiliation**	**Johns Hopkins University Bloomberg School of Public Health**
**Date**	**02-May-2026**
**COI**	**None**

Thank you for the opportunity to review this manuscript. The authors present a protocol for a systematic review focusing on the mental health of Afghan refugees living in high-income countries. This is an important area of inquiry, and the protocol shows promise. At the same time, several aspects of the review's framing and methodology would benefit from additional clarification and justification. Strengthening these areas would help sharpen the study's focus and enhance its contribution. I hope the following comments are helpful as the authors continue to refine the manuscript.

1. Scope of the review

The objectives and research questions of the systematic review are described on page 6; however, they are not clearly articulated and do not appear to align fully.

1) It is unclear whether the authors aim to identify only the types of reported mental health conditions or both the types and their prevalence. Clarifying this distinction is important, as pursuing both would substantially strengthen the contribution of the review.

2) The research questions related to facilitators and barriers (particularly research question #3) lack conceptual clarity. The term “factors that support mental wellbeing” is vague and insufficiently defined. It is unclear whether this refers to individual coping strategies, social support mechanisms, structural determinants, or broader protective factors. Greater specificity would improve interpretability.
3) Furthermore, determinants of mental health and facilitators/barriers to mental health service utilization (research question #2) represent distinct constructs. However, this distinction is not clearly reflected in the research aims, which may lead to conceptual overlap and ambiguity in analysis.
4) A recent systematic review (Hosseini et al., 2023, *Journal of Cross-Cultural Psychology*) examined mental health interventions for Afghan refugees. Although not restricted to high-income settings, most included studies were conducted in such contexts. It would be helpful for the authors to clarify how the proposed review differs from or builds upon this prior work. For example, is the intention to update the literature, refine the scope, or address a distinct gap?

2. Framing in the Introduction

The Introduction appropriately moves beyond individual-level determinants (e.g., trauma, migration stress) and incorporates structural perspectives, including historical and geopolitical factors. However, the current framing appears somewhat one-directional.

While the authors highlight the role of US-led intervention and broader global power dynamics, other relevant contextual factors—such as violence, repression, and governance under the Taliban—are not equally acknowledged. A more balanced framing that considers multiple, potentially overlapping drivers of displacement and mental health outcomes would strengthen the analysis.

3. Search strategy

The search strategy would benefit from improved conceptual precision. Terms such as experiences, perceptions, feelings, attitudes, and perspectives are not mental health conditions; rather, they represent constructs related to lived experience. Combining these directly with clinical mental health conditions using “or” may reduce the specificity of the search.

As currently structured, the search strategy may retrieve studies unrelated to mental health (e.g., Afghan refugees’ perspectives on vaccination or sexual and reproductive health). If the authors intend to capture experiential dimensions of mental health, it would be more

appropriate to distinguish between (1) mental health conditions and (2) related psychosocial or experiential constructs, and structure the search strategy accordingly (e.g., combining domains rather than listing all terms equivalently).

Overall, this systematic review has the potential to generate valuable insights into the mental health of Afghan refugees. However, the manuscript would benefit from clearer and more specific research questions, improved conceptual distinctions, and a more precise search strategy. Addressing these issues would strengthen the rigor and clarity of the review.

VERSION 1 - AUTHOR RESPONSE

Reviewer 1	Response	Edits in manuscript
**Scope of the review**<br>The objectives and research questions of the systematic review are described on page 6; however, they are not clearly articulated and do not appear to align fully.<br><br>1. It is unclear whether the authors aim to identify only the types of reported mental health conditions or both the types and their prevalence. Clarifying this distinction is important, as pursuing both would substantially strengthen the contribution of the review.	Thank you for your comments. We agree that the original objective and research questions needed clearer alignment. We have revised the objective and research questions to separate five areas: reported mental health conditions and prevalence estimates; risk factors for poor mental health; protective factors for wellbeing and resilience; barriers to and enablers of mental healthcare access and use; and interventions designed to improve mental health outcomes.	Page 6-7 'Research questions'<br><br>Pages 6-7
2. The research questions related to facilitators and barriers (particularly research question #3) lack conceptual clarity. The term "factors that support mental wellbeing" is vague and insufficiently defined. It is unclear whether this refers to individual coping strategies, social support mechanisms, structural determinants, or broader protective factors.	Thank you. We have revised the wording to avoid the vague phrase "factors that support mental wellbeing". The manuscript now refers to protective factors at individual, interpersonal, community, service and structural levels.	Page 6

Greater specificity would improve interpretability.		
3. Furthermore, determinants of mental health and facilitators/barriers to mental health service utilization (research question #2) represent distinct constructs. However, this distinction is not clearly reflected in the research aims, which may lead to conceptual overlap and ambiguity in analysis.	As suggested, we have separated determinants of mental health from barriers to and enablers of mental healthcare access and use, as these are related but distinct constructs.	Page 5
4. A recent systematic review (Hosseini et al., 2023, Journal of Cross-Cultural Psychology) examined mental health interventions for Afghan refugees. Although not restricted to high-income settings, most included studies were conducted in such contexts. It would be helpful for the authors to clarify how the proposed review differs from or builds upon this prior work. For example, is the intention to update the literature, refine the scope, or address a distinct gap?	Thank you. We have added text to clarify how this review differs from Hosseini et al (reference 33). Their review focused on evidence-based mental health interventions for Afghan refugees, using cultural adaptation analysis. Our review has a broader scope, including reported mental health conditions, prevalence estimates, risk and protective factors, mental healthcare access, and intervention evidence in high income settings. Additionally, there have been several new studies published since 2023, indicating a need for an updated review of the evidence base. The proposed review therefore updates and extends the existing evidence base rather than duplicating it.<br>We have now clarified this in the introduction.	Pages 5-6
**Framing in the Introduction**<br>The Introduction appropriately moves beyond individual-level determinants (e.g., trauma, migration stress) and incorporates structural perspectives, including historical and geopolitical factors. However, the current framing appears somewhat one-directional. While the authors highlight the role of US-led intervention and broader global power dynamics, other relevant contextual factors—such as violence, repression, and governance under the Taliban—are not equally	Thank you for this suggestion. The Introduction already referred to the systematic exclusion of marginalised groups by Afghanistan's de facto authorities. We have now revised the Introduction to give a fuller and more balanced account of overlapping drivers of displacement and mental health risk, including armed	Page 9

Commented [SD1]: Please see manuscript -- not sure how to further clarify this within the research question. I considered introducing the language of "protective factors across socioecological levels," but wasn't sure if that would serve to further complicate the aims.

acknowledged. A more balanced framing that considers multiple, potentially overlapping drivers of displacement and mental health outcomes would strengthen the analysis.	conflict, Taliban governance, repression, gender-based discriminatory policies, and post-resettlement conditions in host countries.	
**Search strategy**<br>The search strategy would benefit from improved conceptual precision. Terms such as experiences, perceptions, feelings, attitudes, and perspectives are not mental health conditions; rather, they represent constructs related to lived experience. Combining these directly with clinical mental health conditions using "or" may reduce the specificity of the search. As currently structured, the search strategy may retrieve studies unrelated to mental health (e.g., Afghan refugees' perspectives on vaccination or sexual and reproductive health). If the authors intend to capture experiential dimensions of mental health, it would be more appropriate to distinguish between (1) mental health conditions and (2) related psychosocial or experiential constructs, and structure the search strategy accordingly (e.g., combining domains rather than listing all terms equivalently).	Thank you. We agree that terms such as experiences, perceptions and attitudes are not mental health conditions. We have revised the Search strategy section to make clear that the mental health domain includes two subgroups: clinical/symptom-related terms and experiential/service-access terms. The latter were included to capture qualitative and mixed-methods studies on lived experience, distress, wellbeing and mental healthcare access that may not use diagnostic terms in titles or abstracts. We have clarified that irrelevant records, such as studies where experiences or perceptions are unrelated to mental health or mental healthcare, will be excluded during duplicate screening.	
Overall, this systematic review has the potential to generate valuable insights into the mental health of Afghan refugees. However, the manuscript would benefit from clearer and more specific research questions, improved conceptual distinctions, and a more precise search strategy. Addressing these issues would strengthen the rigor and clarity of the review	We sincerely thank the reviewer for their detailed, constructive and thoughtful suggestions. As suggested, we have revised the research questions, clarified the distinction between mental health risk/protective factors and barriers/enablers to service use, and refined the description of the search strategy. We hope these revisions have addressed the issues raised.	

Commented [SD2]: Unsure how to address this comment as the search process is already complete. Should we provide justification for the strategy as it's currently written?

Commented [MSR3R2]: We don't need to redo the search. We have clarified that irrelevant results will be removed. It is always better to have a broader search and narrow down later than the other way round.
